# Rap1 GTPase Activation and Barrier Enhancement in RPE Inhibits Choroidal Neovascularization *In Vivo*

**DOI:** 10.1371/journal.pone.0073070

**Published:** 2013-09-10

**Authors:** Erika S. Wittchen, Eiichi Nishimura, Manabu McCloskey, Haibo Wang, Lawrence A. Quilliam, Magdalena Chrzanowska-Wodnicka, M. Elizabeth Hartnett

**Affiliations:** 1 Department of Cell Biology and Physiology, University of North Carolina, Chapel Hill, North Carolina, United States of America; 2 Moran Eye Center, University of Utah, Salt Lake City, Utah, United States of America; 3 Department of Biochemistry and Molecular Biology, Indiana University, Indianapolis, Indiana, United States of America; 4 Blood Research Institute, Blood Center of Wisconsin, Milwaukee, Wisconsin, United States of America; 5 Department of Ophthalmology, Showa University, Tokyo, Japan; Cedars-Sinai Medical Center, United States of America

## Abstract

Loss of barrier integrity precedes the development of pathologies such as metastasis, inflammatory disorders, and blood-retinal barrier breakdown present in neovascular age-related macular degeneration. Rap1 GTPase is involved in regulating both endothelial and epithelial cell junctions; the specific role of Rap1A vs. Rap1B isoforms is less clear. Compromise of retinal pigment epithelium barrier function is a contributing factor to the development of AMD. We utilized shRNA of Rap1 isoforms in cultured human retinal pigment epithelial cells, along with knockout mouse models to test the role of Rap1 on promoting RPE barrier properties, with emphasis on the dynamic junctional regulation that is triggered when the adhesion between cells is challenged. *In vitro*, Rap1A shRNA reduced steady-state barrier integrity, whereas Rap1B shRNA affected dynamic junctional responses. In a laser-induced choroidal neovascularization (CNV) model of macular degeneration, *Rap1b^−/−^* mice exhibited larger CNV volumes compared to wild-type or *Rap1a^−/−^*. *In vivo*, intravitreal injection of a cAMP analog (8CPT-2′-O-Me-cAMP) that is a known Rap1 activator significantly reduced laser-induced CNV volume, which correlated with the inhibition of CEC transmigration across 8CPT-2′O-Me-cAMP-treated RPE monolayers *in vitro*. Rap1 activation by 8CPT-2′-O-Me-cAMP treatment increased recruitment of junctional proteins and F-actin to cell-cell contacts, increasing both the linearity of junctions *in vitro* and in cells surrounding laser-induced lesions *in vivo*. We conclude that *in vitro*, Rap1A may be important for steady state barrier integrity, while Rap1B is involved more in dynamic junctional responses such as resistance to junctional disassembly induced by EGTA and reassembly of cell junctions following disruption. Furthermore, activation of Rap1 *in vivo* inhibited development of choroidal neovascular lesions in a laser-injury model. Our data suggest that targeting Rap1 isoforms *in vivo* with 8CPT-2′-O-Me-cAMP may be a viable pharmacological means to strengthen the RPE barrier against the pathological choroidal endothelial cell invasion that occurs in macular degeneration.

## Introduction

The barriers created by epithelial and endothelial cell sheets in the body are critical to maintain physiological homeostasis by functioning to limit movement of fluids, solutes, macromolecules, and the passage of other cells or pathogens from one side of a monolayer to the other. The blood-brain and blood-retinal barriers are extreme examples of this tight regulation. If the integrity of the endothelial or epithelial barrier is compromised, hyper-permeability, edema, inappropriate inflammation, and invasion of non-resident cells can occur; this can lead to pathologies in stroke and cardiovascular disease, autoimmune disorders, tumor metastasis, and ocular diseases, including diabetic retinopathy, retinal vein occlusion and age-related macular degeneration (AMD).

Tight junctions and adherens junctions are sites of adhesion between adjacent cells, and the transmembrane protein components of these structures comprise the physical barrier of the paracellular pathway. Transmembrane proteins, such as occludin, members of the claudin family, and cadherins, also act as protein scaffolds for cytoplasmic proteins such as ZO-1, β-, α-, and p120- catenin, some of which bind to the actin cytoskeleton [1]. This linkage between junctional complexes and the F-actin cytoskeleton is critical for the dynamic opening and resealing of junctions, and is necessary to allow rapid responses to cellular events. Furthermore, junctional adhesion may be strengthened to resist insult, and/or repaired in response to challenge or injury. Of the signaling proteins involved in junctional regulation, small GTPases are particularly well-suited to rapid fine-tuning of barrier integrity owing to their ability to cycle between active (GTP-bound) and inactive (GDP-bound) states. Small GTPases of the Rho family are regulators of cell junctions [2], [3]; how this occurs relates to the ability of Rho GTPase signaling to affect actin cytoskeleton remodeling [4].

In addition to the GTPases of the Rho family, we have also become interested in another GTPase, Rap1, which is a member of the Ras superfamily [5]. In addition to its role in integrin-mediated cell matrix adhesion and cell migration [6], Rap1 has been shown by many groups to regulate cell junctional integrity, and barrier function of endothelial and epithelial cell monolayers [7]–[13]; Rap1-induced junctional strengthening has been shown to inhibit monocyte transendothelial migration [9]. There are two isoforms of Rap1, Rap1A and Rap1B. Whereas Rap1a and Rap1b knockout mice are each viable and fertile [14], [15], the double Rap1a/Rap1b knockout is lethal [16]. While Rap1a-null mice were originally found to develop normally with no gross phenotypic abnormalities [15], [17], subsequent backcross into C57Bl/6J background produced some (∼40%) embryonic lethality associated with cardiac defects (J. Yan and L. Quilliam, unpublished data). Rap1b-null mice exhibit 85% perinatal lethality, likely due to complications arising from embryonic hemorrhage [14]. Rap1A and Rap1B isoforms are on different chromosomes, but are 94% identical, with only 9 amino acids different between them, and oftentimes the literature has not explicitly distinguished between the two. Recently, using *in vitro* shRNA experiments in human endothelial cells, Rap1A was shown to be the isoform important for attaining mature, steady state junctional barrier properties [13].

The retinal pigment epithelium (RPE) makes up the outer blood retinal barrier between the retina and the overlying sensory retina. Breakdown of this barrier is a step in the pathogenesis of neovascular AMD [18], the leading cause of central vision loss world-wide in patients over the age of 60 years [19]. In neovascular AMD, choroidal endothelial cells (CECs) are activated to migrate to, and across the RPE monolayer, leading to the development of vision-impairing choroidal neovascularization (CNV) in the sensory retina. As such, therapies in the form of angiogenic inhibitors, including those acting against the bioactivity of vascular endothelial growth factor (VEGF), have been the mainstay of AMD treatment [20]; however, because VEGF is also a neuroprotective agent [21] and a survival factor for endothelial cells [22], inhibiting its bioactivity may not only inhibit CNV, but also reduce these beneficial properties of VEGF. Therefore, methods other than inhibition of angiogenesis to treat neovascular AMD are under investigation, such as factors that regulate RPE barrier integrity and prevent damaging CEC transmigration into the sensory retina.

## Materials and Methods

### Ethics statement

#### Animal care and use

All animals were cared for in accordance with the Association for Research in Vision and Ophthalmology Statement for the Use of Animals in Ophthalmic and Vision Research; all animal use protocols were approved by the Institutional Animal Care and Use Committee of the University of Utah (IACUC protocol #10–06002), and of the University of North Carolina (IACUC protocol #10–169).

#### Human samples

Primary human choroidal ECs (CECs) were isolated from donor eyes (existing pathological specimens) obtained from the North Carolina Eye Bank (http://www.nceyebank.org) or the Utah Lions Eye Bank (http://www.utaheyebank.org) as described previously [23].

### Cell Culture

ARPE-19 cells (RPE) were obtained from ATCC (Rockville, MD), grown in DMEM/F-12 plus 10% FBS, and used from passage 15–20 when epithelioid properties are still present [24]. CECs were grown in endothelial growth media (EGM-2; Lonza, Walkersville, MD) supplemented with 10% FBS and used from passage 2–4.

### shRNA of Rap1A and Rap1B

Adenoviral constructs encoding microRNAs designed to specifically target and knockdown expression of Rap1A or Rap1B have been described previously [13]. Negative control shRNA adenovirus expresses an insert that is processed into a mature shRNA, but it is not predicted to target any known vertebrate gene. In ARPE-19 cells, efficient knockdown was attained 48 to 72 hours post-virus addition. Confirmation of knockdown was obtained by Western blot analysis of cell lysates using primary antibodies that specifically recognize Rap1A or Rap1B isoforms (anti-Rap1A rabbit pAb SC-1482R, Santa Cruz; anti-Rap1B rabbit mAb #2326S, Cell Signaling Technology), or an antibody that recognizes both isoforms (A+B) (Santa Cruz #sc-65),. Equal gel loading was confirmed by probing with anti-β-actin (MAB1501, Chemicon).

### Calcium Switch Assays

The “calcium switch” assay is a method for assessing cell junctional disassembly and reassembly, using EGTA treatment and subsequent washout as a means of inducing dynamic changes in barrier function that can be measured experimentally [25]. For calcium switch experiments, ARPE-19 cells were cultured on coverslips, and then transduced with negative control, Rap1A, or Rap1B shRNA construct for another 3 days before performing the calcium switch assay. EGTA (4 mM) was added for 5–30 minutes to disrupt junctions; washout with calcium-containing cell growth media for 0.5–24 hr stimulated synchronous junctional resealing.

### Analysis of Monolayer Gaps

At the indicated time points of EGTA addition or washout, ARPE-19 cell monolayers were fixed and stained as described previously [26]. Fluorescence images were obtained with an Axiovert 200 M microscope (Zeiss,) equipped with a digital camera (Hamamatsu ORCA-ERAG) and acquired using Metamorph (Universal Imaging Corp.). To quantify RPE monolayer integrity, images taken from multiple random fields were analyzed in ImageJ by thresholding and using the “area fraction measurement” tool to obtain the number of pixels representing the area not covered by cells, and expressed as percent gap area of the total area of the image field. Prior to image analysis, file names were randomized to ensure unbiased analysis. Cell area was readily identified by cytoplasmic fluorescence from the cocistronically expressed EmGFP contained within the shRNA expression cassette.

### Barrier Function Assays

#### Transepithelial Electrical Resistance (TER)

ARPE-19 were plated at confluent density (1.8×10^5^ cells) on 12 mm diameter, 0.4 μm pore size Transwell filters (in at least quadruplicate). TER was measured at given time points using an Endohm-12 Transwell Chamber connected to an EVOM Voltohmmeter according to the manufacturer's instructions. “Day 0” TER readings were taken immediately before shRNA virus addition; steady state TER was measured again at day 5 post-infection. Data for these experiments are expressed as normalized TER relative to the initial day 0 value (average from n = 3 independent experiments).

#### Real-time cell analysis (RTCA) experiments

Barrier properties of RPE were also measured using the xCELLigence Real-Time Cell Analyzer (RTCA) system (Acea Biosciences/Roche Applied Science). This technology measures electrical impedance as a readout for the barrier status of cells grown directly on biocompatible micro-electrode coated surfaces. Changes in impedance (represented as “Normalized Cell Impedance”) reflect changes in barrier function and permeability [27], [28]. ARPE-19 treated with indicated shRNA were grown on the microelectrode-coated wells of an E-Plate 16 (Roche Applied Science), and then treated with 4 mM EGTA to induce barrier breakdown. Monolayer impedance was recorded at 1 min intervals for 90 min in the presence of EGTA. A representative trace from 3 independent experiments is shown, graphed as average of quadruplicate wells ± SD.

### CEC Transmigration Assay

CEC transmigration across the RPE monolayer was measured as previously described with minor modification [23]. Briefly, ARPE-19 cells were plated onto the underside of 8 μm pore size Transwells (Costar/Corning), and were treated with 250 µM 8CPT-2′O-Me-cAMP (Biolog) overnight. Primary human CECs were fluorescently-labeled (VybrantDio Cell-labeling solution; Invitrogen), and then plated in the top well of the inserts. After 48 h, CECs which had migrated across the filter and the RPE, were counted per 20× field by fluorescence microscopy. Multiple random fields were quantified, averaged, and data presented as mean ± SEM from 6 Transwells per condition.

### *In vivo* Laser-induced CNV Model

Wild type (WT) C57/Bl6 mice were litter-mate controls from Rap1a or Rap1b knockout mice. *Rap1a^−/−^* and *Rap1b^−/−^* mice were as described [14], [29]. Mice aged between 11–20 weeks were used for these studies.

#### Laser treatment

The laser-induced choroidal neovascularization (CNV) model was carried out as previously described [30]. Briefly, 11–20 week old mice were anesthetized then raised on a platform to the slit lamp (30 SL-M, Carl Zeiss Meditec Inc. CA) to perform laser photocoagulation (150 mW, 100 ms, 100 μm) using a 532-nm OcuLight GL laser (Iridex, Irvine, CA).

#### RPE/Choroid Flat mount

Eyes were enucleated then fixed in 4% paraformaldehyde/PBS for 1 hr, then posterior eyecups consisting of the RPE/choroid/sclera were dissected out. Eyecups were treated with 0.1% Triton X-100 before labeling for indicated junctional proteins. Primary antibodies: anti-β-catenin (pAb C2206, Sigma), anti-ZO-1 (mAb clone1A12, Invitrogen); F-actin was visualized using Texas Red-conjugated phalloidin and nuclei by Hoechst 33342 (Invitrogen). For visualization of laser lesions, eyecups were stained with AlexaFluor 568-conjugated isolectin B4 (Invitrogen, CA) overnight at 4°C to label invading choroidal vessels. After staining, the eyecups were flattened by cutting radial incisions and flat-mounted onto a microscope slide with Vectashield mounting medium (Vector Laboratories).

#### Quantification of β-catenin junctional localization

Images were analyzed for β-catenin fluorescence intensity at cell-cell contacts using ImageJ (National Institutes of Health) as described in [31]. Briefly, mean pixel intensity of immunolabeled β-catenin was measured in a small (25 µm^2^) circular region of interest (ROI) drawn over a cell junction (two opposing cell membranes). For each measurement, cytoplasmic background was subtracted by moving the circular ROI to a region immediately adjacent to the measured junction. A total of 100 cell-cell junctions were measured per condition.

#### Confocal Imaging and Quantification of Lesion Volume

Flat mounts were imaged by taking optical Z-sections at 1 µm increments with a confocal microscope (Olympus FV1000) and summing areas to create a volume. Confocal stacks were analyzed with 3D image-analysis software (Volocity 5.0; Improvision/PerkinElmer) to obtain each isolectin-positive lesion volume (µm^3^). Lesions with obvious hemorrhage or bridging CNV were excluded. For experiments comparing WT and knockout mice, 17–26 lesions, from at least 6 mice per genotype were analyzed. For 8CPT-cAMP dose experiment, 13–21 lesions from at least 4 mice injected with each dose were analyzed.

### *In Vivo* Rap1 activation with 8CPT-cAMP

Intravitreal injection was done using a MICROLITER^TM^ syringe (Hamilton Company) immediately after laser administration. 8CPT-cAMP was injected at doses that have been shown to activate Rap1, but not PKA (i.e. <100 µM, [32]), each in a volume of 1 µl. Phosphate buffer saline (PBS) injection was used as the control.

#### Rap1 Activity Assays

Three to four hours following laser injury and intravitreal injection of 8CPT-cAMP (2.05 µM) or PBS, RPE/choroids were isolated from euthanized mice, and lysed and homogenized by sonication in RIPA buffer. Supernatants were used for Western blotting using an antibody specific for active (GTP-bound) Rap1 (NewEast, PA), and β-actin (Santa Cruz, CA). Digitized images of Western blots were quantified using UN-SCAN-IT gel 6.1 (Silk Scientific) and normalized to β-actin.

#### Quantification of Junctional Linearity

A “junctional linearity index”, defined as the ratio of actual junction length to the linear junction length (straight line distance between vertices) was calculated as described [33]. Cell junctions were manually traced and measured using ImageJ. The closer the value to “1”, the higher the degree of linearity.

### *In Vivo* Toxicity Assays

#### Retinal Thickness

Following laser and vitreous injection of 8CPT and PBS, retinal thickness measurements were taken with a spectral-domain optical coherence tomography unit (sd-OCT; Bioptogen, NC), in 2–4 discrete locations in each eye. Locations were 2 optic nerve disc diameters from the optic nerve and avoided areas encompassing laser lesions. Retinal thicknesses of 18 eyes for each condition were obtained to compare 8CPT vs. PBS injection, and image analysis was done with InVivoVue Clinic software.

#### Cleaved-caspase3 assay

24 hr following administration of 8CPT-cAMP or PBS, mice were euthanized and the RPE/choroids were isolated, lysed in RIPA buffer, and homogenized using sonication. Supernatants were collected and blotted for cleaved caspase-3 (Cell Signaling, MA) and for total caspase-3. As a positive control for apoptosis, H1B-1B cells were treated with staurosporine (1 µM, 3 hr at 37°C), and then blotted for cleaved caspase-3.

#### TUNEL Staining

Eyes were fixed in 4% PFA, incubated in 15% sucrose until sinking, and then incubated in 30% sucrose overnight at 4°C. Samples were then embedded in O.C.T. Compound, frozen rapidly with dry ice, and stored at −80°C until sectioning. Cryosections with thicknesses of 10 to 12 µm were prepared using a cryostat (CM3050S, Leica, NJ). Apoptotic cells were visualized by TUNEL using the In Situ Cell Death Kit-Fluorescein (Roche Diagnostics, Germany) according to manufacturer's protocol. DNase-treated cryosections (Fermentas-EN0521; 0.33 U/µl in PBS, 30 min) served as a positive control for TUNEL assay.

### Statistical Analysis

#### *In vitro* experiments

Statistical significance was determined by Student's *t*-test (one-tail, equal variance) using the average values ± SEM obtained from at least triplicate samples in a given experiment, or normalized pooled data from at least 3 independent experiments, unless noted in the figure legend. A *P* value of <0.05 was considered statistically significant. Immunofluorescence data, including confocal imaging and Western blot analyses are representative of multiple independent experiments.

#### *In vivo* experiments

Mean CNV volume of lesions for *Rap1a^−/−^* and *Rap1b^−/−^* mice, each dose of 8CPT-cAMP, and control were analyzed by Kruskal-Wallis test. Active Rap1 in RPE/choroids was analyzed by ANOVA. Post-hoc testing was performed using the Fisher's LSD test for parametric tests and the Steel-Dwass test for non- parametric tests. An alpha level of <0.01 was used as criterion of significance.

## Results

**Figure 1:**
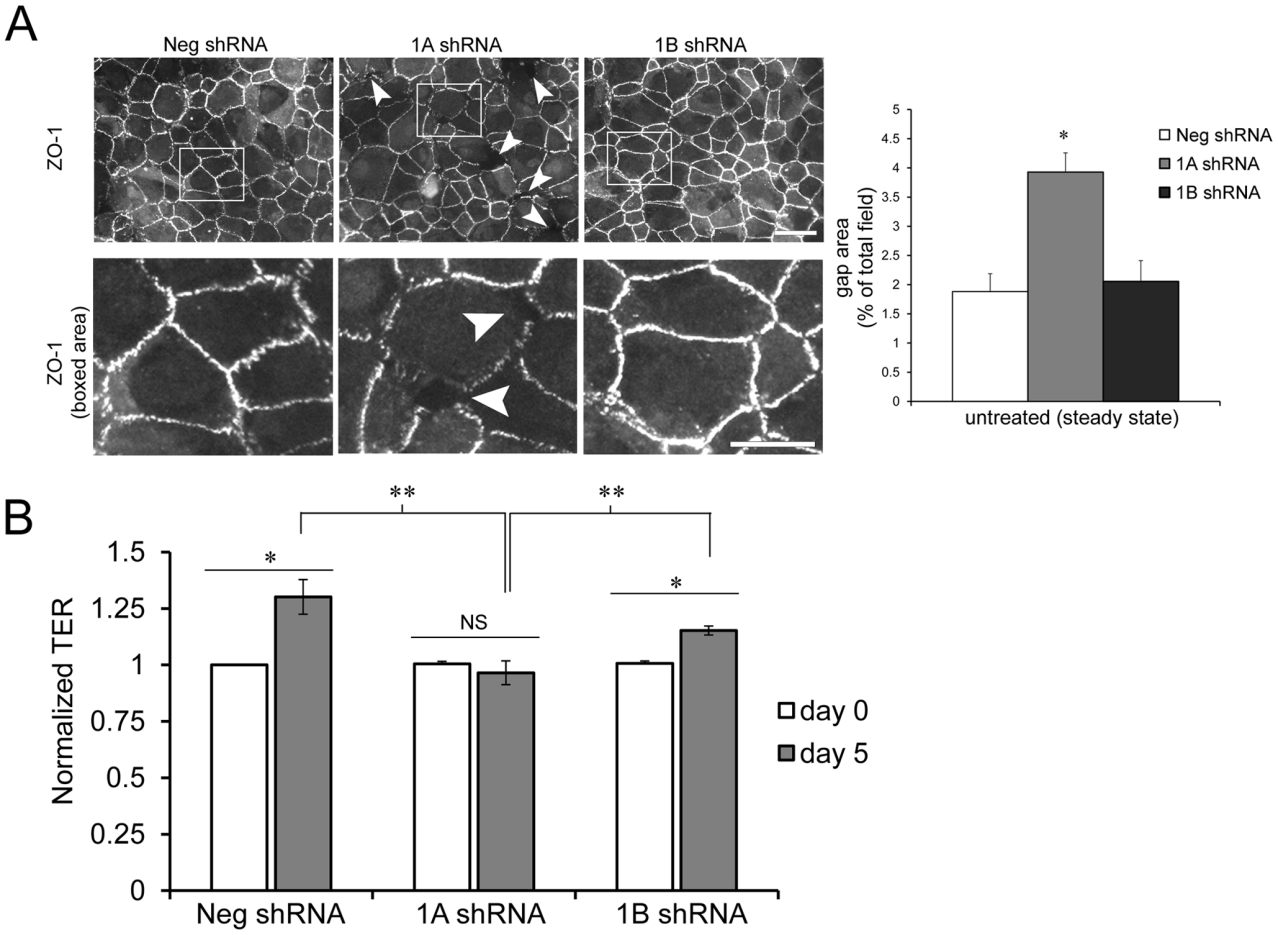
Rap1A knockdown *in vitro* impairs steady state monolayer integrity and decreases transepithelial electrical resistance. (A) RPE cells transduced with indicated shRNA constructs were grown on coverslips for 4–5 days to obtain steady state monolayers. Top row, representative immunofluorescence localization of the junctional marker ZO-1. Scale bar  = 50 µm. Boxed areas are enlarged to highlight intercellular gaps in Rap1A shRNA cell monolayers (bottom row). Scale bar  = 25 µm. Quantification of total monolayer gap area per image field (expressed as % of total field area) using image analysis software (ImageJ) was used as an indication of monolayer integrity. Graph shows the mean of n = 4 fields ± SEM, representative experiment from 2 independent trials. **p<0.01, 1A shRNA compared to both Neg and 1B shRNA. (B) TER was measured on cells prior to shRNA knockdown (day 0), and again after 5 days. Graph shows TER of negative control, Rap1A, or Rap1B shRNA treated cells, representing average TER normalized to control at t = day 0 from n = 4 independent experiments. Control shRNA and Rap1B shRNA cells increased TER after 5 days in culture, while Rap1A shRNA cells did not significantly increase TER from day 0 to day 5. * p≤0.01, NS =  not significant; **p≤0.01, TER of Rap1A shRNA monolayers significantly lower at steady state (day 5) compared to control and Rap1B shRNA.

While previous work has implicated Rap1 in cell junction regulation, information is still lacking on isoform-specific functions for Rap1A and Rap1B, both during maintenance of mature junctions at steady state and during the dynamic junctional regulation that must occur during assembly and disassembly. We treated RPE cells *in vitro* with Rap1 isoform-specific shRNA constructs [13], [26], and assessed monolayer integrity and barrier function under both of these conditions. Confirmation of knockdown and antibody specificity is shown in Fig. S1. shRNA-treated RPE were plated for 4–5 days to allow stable cell monolayers to develop. Representative immunofluorescent images of ZO-1 localization at steady state (Fig. 1A) show that control and Rap1B shRNA monolayers exhibit a continuous junctional ZO-1 staining pattern with very few gaps between cells. By contrast, Rap1A shRNA RPE seemed to have more discontinuous ZO-1 staining and greater gaps between cells in the monolayer (arrowheads). Monolayer integrity was quantified using image analysis software to measure the average gap area of each shRNA monolayer from multiple random fields (Fig. 1A, graph). Rap1A shRNA-RPE monolayers had significantly greater gap area than either control or Rap1B shRNA monolayers, indicating that at steady state, Rap1A is important for maintenance of a confluent, gap-free monolayer. We also examined these shRNA-treated RPE using transepithelial electrical resistance (TER) as a read-out of monolayer barrier function. TER was measured before indicated shRNA-treatments (“day 0”) and again after 5 days (Fig. 1B). Both negative control and Rap1B shRNA-treated RPE monolayers had significantly higher TER at day 5 compared to day 0; however, the TER of Rap1A shRNA-treated RPE did not increase significantly over the same time period. Steady state TER after 5 days was significantly lower in Rap1A shRNA monolayers compared to control and Rap1B shRNA. The observations that Rap1A shRNA increased monolayer gap area and decreased TER imply that Rap1A may be more important for acquisition of steady state TER.

**Figure 2:**
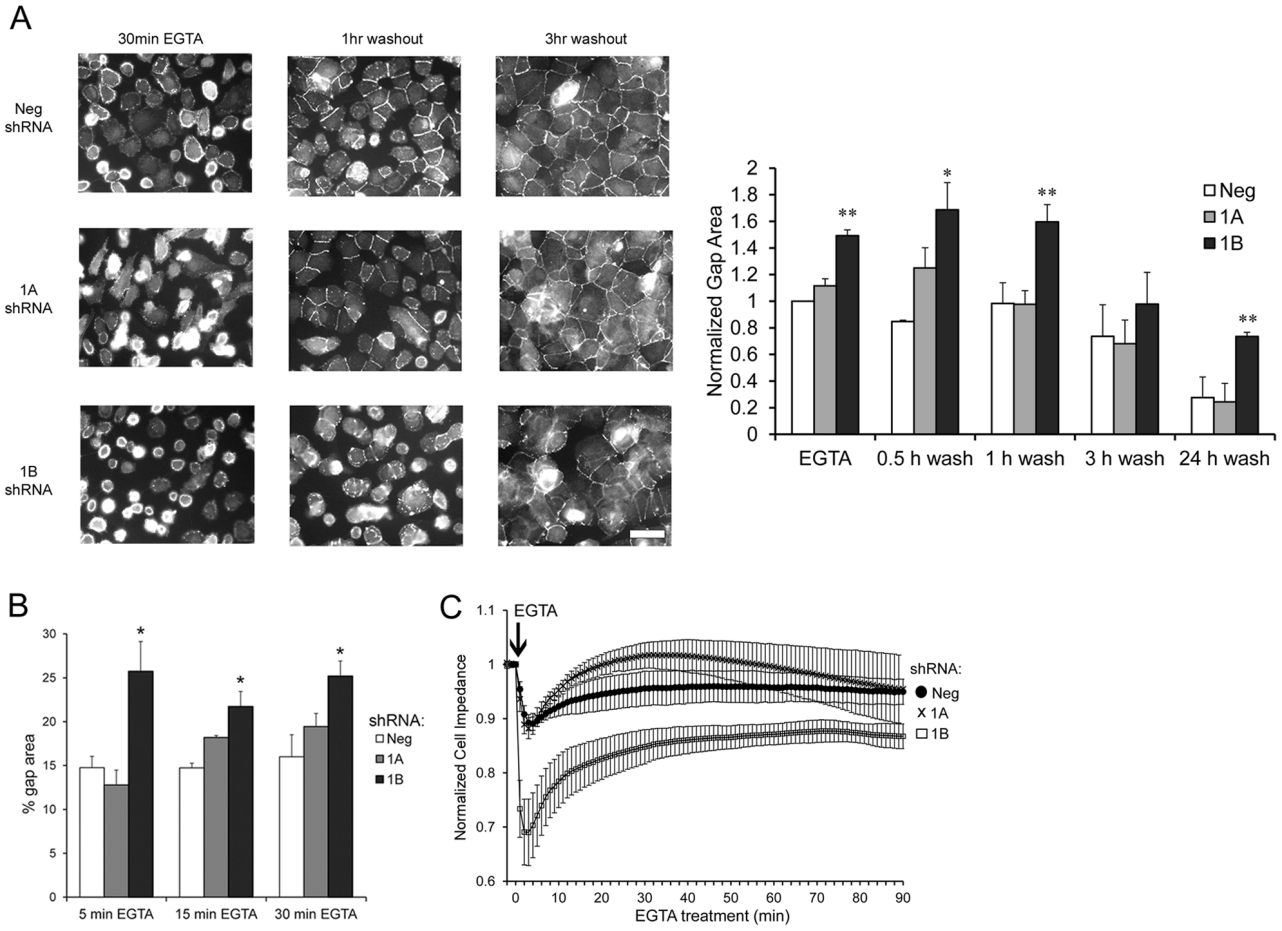
Rap1B knockdown *in vitro* affects dynamic junctional responses. Monolayer re-formation and resistance to EGTA-induced junctional disassembly were assessed using the calcium switch assay. (A) Representative immunofluorescence localization of ZO-1 after 30 min EGTA to disrupt junctions (left column), followed by 1 or 3 hr of washout to synchronously initiate junctional re-assembly (middle and right columns). Note greater cell-free areas (gaps) and impaired recruitment of ZO-1 to cell-cell contacts in Rap1B shRNA RPE. Quantification of the monolayer gap area per image field is shown in the graph. Recovery of Rap1B shRNA monolayer integrity was delayed compared to Neg control and Rap1A shRNA. (* compared to Neg, ** compared to Neg and Rap1A shRNA). Pooled averages (multiple random fields) from n = 3 independent experiments ± SEM, normalized to gap area of Neg control monolayers after EGTA treatment. *, **p≤0.05 (B) Gap area during EGTA-induced junctional disassembly was quantified as in panel A. EGTA treatment caused larger gaps in Rap1B shRNA monolayers compared with control or Rap1A shRNA monolayers. Graph shows the mean of n = 4 fields ± SEM, representative experiment from 2 independent trials. *p≤0.05, 1B shRNA compared to both Neg and 1A shRNA. (C) Electrical impedance analysis of cell monolayer disruption induced by EGTA. Data points represent the average ± SD from quadruplicate wells for each condition. Representative trace of 3 independent experiments.

To examine RPE cell junctions under dynamic disassembly/reassembly conditions, we also subjected shRNA-treated RPE monolayers to a calcium switch protocol as described in the Materials and Methods. Representative immunofluorescent images of ZO-1 localization in negative control vs. Rap1A or Rap1B shRNA cells subjected to calcium switch are shown in Fig. 2A and Fig. S2. While all cell monolayers exhibited loss of junctional ZO-1 staining along with cell retraction and appearance of monolayer gaps after 30 min EGTA treatment, quantification of the gap area (graph at right) revealed that Rap1B shRNA caused significantly greater monolayer disruption (greater gap area). After 1–3 hr EGTA washout, junctions of the control and Rap1A shRNA monolayer were beginning to reseal, with the recruitment of ZO-1 becoming apparent and the monolayer gap size decreasing. In contrast, Rap1B shRNA cell monolayers show delayed reassembly of cell junctions, as evidenced by incomplete junctional relocalization of ZO-1 after a 3 hr washout and the persistence of monolayer gaps even after 24 hr washout. We also looked at junctional disassembly by treating cell monolayers to an EGTA time course and quantifying monolayer gap area (Fig. 2B). At each time point, Rap1B-shRNA RPE monolayers had significantly greater gap area than control or Rap1A shRNA-treated monolayers. Finally, we confirmed that this increase gap area corresponded with barrier function by performing real time cellular analysis of electrical impedance (RTCA) of cell monolayers. shRNA-treated monolayers were grown as a monolayer on micro-electrode coated surfaces, and then EGTA was added (arrow) to induce junctional disassembly (indicated by a decrease in Cell Impedance). Representative impedance trace (Fig. 2C) shows that barrier function, measured by impedance, is decreased more in Rap1B shRNA monolayers treated with EGTA compared to control or Rap1A shRNA monolayers. There was no significant difference between control or Rap1A shRNA treated monolayers. Taken together, these results imply that *in vitro*, loss of Rap1A impairs steady state junctional maintenance under control conditions (Fig. 1A–B), whereas loss of Rap1B affects more dynamic events such as junctional reassembly/disassembly (Fig. 2A–C).

**Figure 3:**
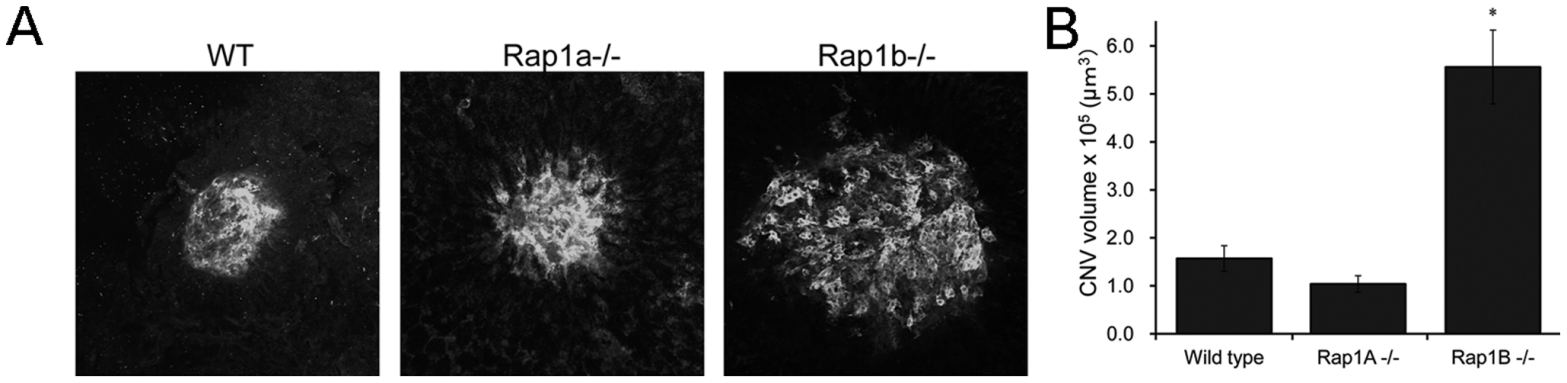
Laser-induced CNV in WT vs. *Rap1a^−/−^* and *Rap1b^−/−^* mice. One week following laser, eyes were processed as RPE/choroid flat mounts and stained with AlexaFluor 568-labeled lectin to visualize the choroidal neovascular endothelial cells. (A) Representative confocal images from WT, *Rap1a^−/−^*, and *Rap1b^−/−^* (maximum projections). (B) Quantification shows that CNV volume is significantly greater in *Rap1b^−/−^* compared to WT and *Rap1a^−/−^* mice. Bars represent average lesion volume ± SEM from at least 6 individual mice per genotype (n = 17–26 lesions). * p<0.01, *Rap1b^−/−^* compared to WT and *Rap1a^−/−^*.

Breakdown of the RPE barrier during neovascular AMD is an *in vivo* example of a situation where impaired junctional resealing would contribute to the pathology. Our in vitro results led us to explore whether Rap1A and/or Rap1B are key to the maintenance of this barrier. We turned to a well-accepted *in vivo* model of choroidal neovascularization induced by laser injury (laser-CNV) [30] to explore whether loss of Rap1 inhibits RPE barrier function and facilitates CNV formation. In this model, the laser dramatically disrupts the RPE monolayer locally where the laser spot is administered. While some RPE cells directly in the laser spot are destroyed, neighboring RPE produce reactive oxygen species that can be measured by dihydroethidium staining [34]. This and other inflammatory and wound-healing events occur that lead to activation of CECs from the choriocapillaris to migrate and proliferate within subretinal space to form CNV lesions. One week post-laser, we analyzed extent of CNV as width of lesion at the RPE layer measured by spectral domain-optical coherence tomography (sd-OCT) and volume by measuring and summing areas axially obtained at 1 µm intervals by confocal microscopy of lectin-stained RPE/choroid flat mounts from WT, *Rap1a^−/−^*, and *Rap1b^−/−^* mice. Representative maximum projections of lectin-stained CNV are shown in Fig. 3A. Quantification of lesion volume revealed that *Rap1b^−/−^* had significantly larger CNV volumes compared to either WT or *Rap1a^−/−^* (Fig. 3B).

**Figure 4:**
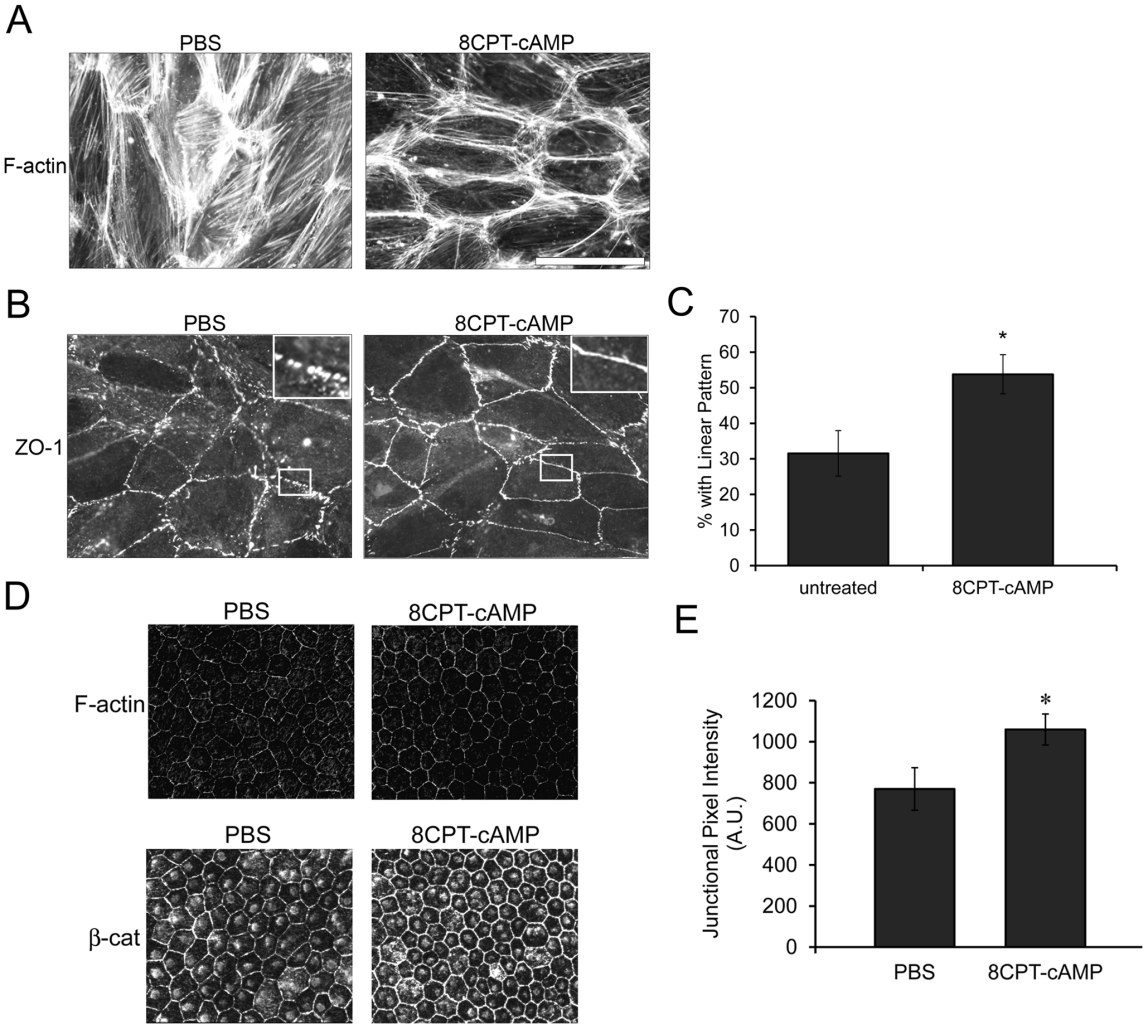
Activation of total Rap1 enhances recruitment of junctional proteins and cortical F-actin both *in vitro* and *in vivo*. (A) 8CPT-cAMP treatment (250 µM, 1 hr) of cultured RPE monolayers decreases stress fibers and enhances cortical F-actin morphology. (B) Enhanced recruitment and linear junctional staining of ZO-1 with 8CPT-cAMP treatment compared to PBS control. Boxed areas are enlarged in the upper right inset to highlight differences in junctional staining pattern. Scale bar, 50 µm (C) Quantification of *in vitro* 8CPT-cAMP treatment. Percent of cells that show enhanced (linear) junctional localization of ZO-1 (average % of 10 fields/condition (>600 cells total counted). * p<0.01 (D) *In vivo*, eyes injected with 8CPT-cAMP also have enhanced linear junctional recruitment of proteins such as F-actin and β-catenin. (E) Quantification of junctional β-catenin in PBS vs. 8CPT-cAMP-injected eyes. Data plotted as average junctional pixel intensity from random cells per field, from n = 4 injected eyes (>100 cells). * p = 0.0324 compared to PBS-injected.

Based on these results, we hypothesized that conversely, CEC transmigration and CNV formation would be reduced by activating Rap1 isoforms in RPE. 8CPT-2′-O-Me-cAMP (8CPT-cAMP) is a cAMP analog that binds to and activates the Rap1 exchange factors Epac1 and Epac2, but does not activate other cAMP-responsive pathways involving PKA and other downstream pathways [32]. In cultured RPE cells, treatment with 250 µM 8CPT-cAMP is sufficient to activate total Rap1 and increase monolayer barrier function (cellular impedance) [26]. Furthermore, Rap1 activation by 8CPT-cAMP treatment of RPE caused stress fiber dissolution, stabilizing the cortical localization of F-actin (Fig. 4A), similar to published reports for other cell types [10], [35]–[38]. ZO-1 was also enriched at cell junctions with the localization appearing more linear and continuous, having fewer cells with punctate discontinuous ZO-1 staining compared to PBS-treated (Fig. 4B, insets upper right). In short-term culture experiments like this, ARPE can exhibit some discontinuous staining at baseline, which we use to represent RPE in “stressed or aged” conditions. When quantified, 8CPT-cAMP treatment was confirmed to significantly enhance the junctional recruitment and linear pattern of ZO-1 compared to PBS-treated control (Fig. 4C). We then asked whether this also occurred *in vivo* upon 8CPT-cAMP intravitreal injection. Fig. 4D shows representative confocal images of flat mounted murine eyes that had been injected with PBS or 8CPT-cAMP. 8CPT-cAMP injection induced a slight increase in F-actin localization to the cell periphery, and reorganized cell junctions to a more linear shape. β-catenin junctional localization was also reorganized after 8CPT-cAMP intravitreal injection. Quantification of β-catenin pixel intensity confirmed a significant increase in junctional localization after 8CPT-cAMP treatment (Fig. 4E).

**Figure 5:**
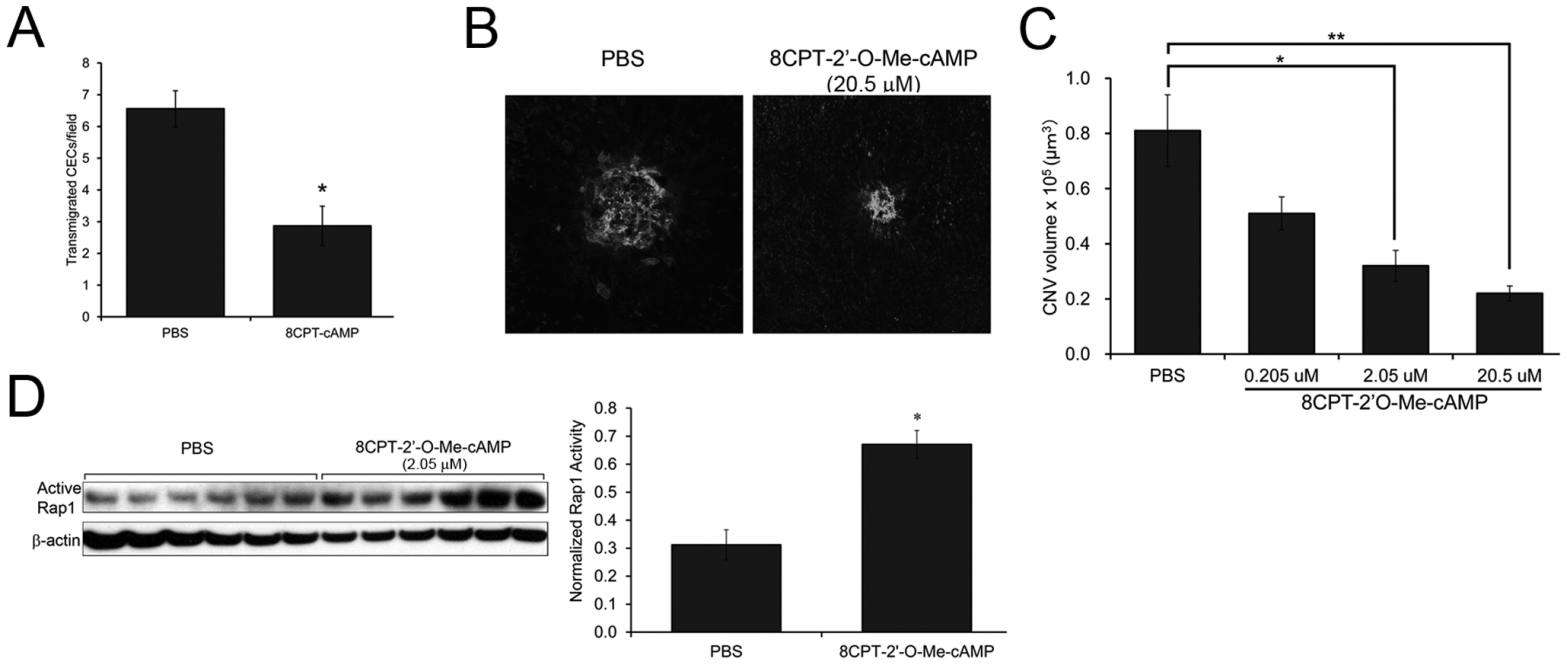
CEC transmigration across RPE monolayer (*in vitro*) and CNV volume (*in vivo*) are inhibited with 8CPT-cAMP treatment. (A) *In vitro*, CECs have decreased transmigration across RPE monolayers when RPE are incubated with 8CPT-CAMP. Representative experiment of 2 independent trials. Data plotted as the average number of transmigrated CECs (± SEM) per 3 random fields, n = 6 Transwells per condition. * p<0.001 (B) Representative confocal images (maximum projections) of lectin-stained RPE/choroid flat mounts, 1 week post laser and intravitreal injection of PBS or 20.5 µM 8CPT-cAMP. (C) Quantification: intravitreal injection of 8CPT-cAMP induces a dose-dependent decrease in CNV volume in compared to PBS-injected control. *p<0.01, **p<0.001 (n = 13–21 lesions per condition) (D) Intravitreal injection of 8CPT-cAMP activates Rap1 *in vivo*. RPE/choroid was dissected and lysates were blotted with an antibody that detects active Rap1 (GTP-bound), or β-actin as loading control. Graph shows densitometry of active Rap1 normalized to β-actin (average ± SEM, n = 6). * p<0.0001.

We then tested whether the barrier enhancing-effects of activating Rap1 with 8CPT-cAMP could protect against CEC transmigration across RPE grown as inverted monolayers on Transwell filters [23], [39]. Compared to the PBS-treated control, 8CPT-cAMP treatment significantly reduced CEC transmigration across the RPE monolayer *in vitro* (Fig. 5A). Next, we tested whether activation of Rap1 *in vivo* would abrogate laser-induced CNV. 8CPT-cAMP at indicated doses was administered by intravitreal injection [40] immediately following laser, and CNV volumes were quantified 1 week later. Representative confocal images of lectin-stained flat mounts of PBS and 20.5 µM 8CPT-cAMP-injected eyes are shown in Fig. 5B. Compared to PBS-injected control, there was a dose-dependent decrease in CNV volume with increasing concentration of drug (Fig. 5C). We confirmed that treatment with 8CPT-cAMP increased Rap1 activity in isolated RPE/choroids following intravitreal injection (Fig. 5D). Furthermore, we tested for toxicity of intravitreal injection of 8CPT-cAMP. There was no significant difference in retinal thickness measured by sd-OCT in PBS vs. 8CPT-cAMP-injected eyes (Fig. S3A–C). In addition, cleaved caspase-3, a cellular marker indicator of increased apoptosis, was not detected in PBS or 8CPT-cAMP-injected eyes 24 hours after injection (Fig. S3D). Lastly, there was negligible amount of positive TUNEL staining of cryosections of injected eyes (Fig. S3E). These measurements all indicate that toxicity of 8CPT-cAMP was low. Combined, these results indicate that activation of both Rap1 isoforms in RPE with 8CPT-cAMP treatment can inhibit transmigration of CECs across the epithelium *in vitro*, and this correlated with decreased CNV volume *in vivo*.

**Figure 6:**
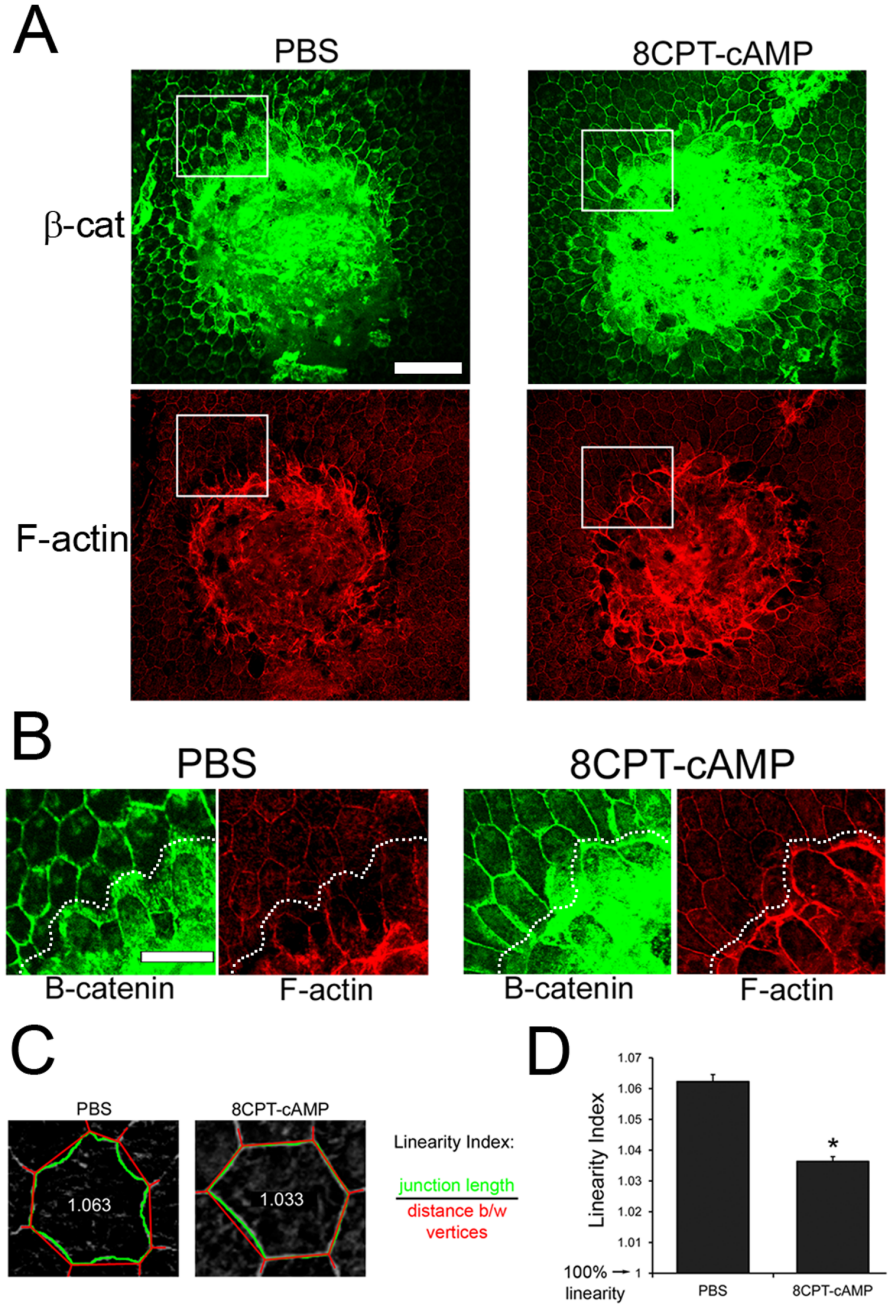
Recruitment of junctional proteins and increased junctional linearity adjacent to lesions. (A) β-catenin and F-actin staining around lesions is enhanced in 8CPT-injected eyes. Scale bar, 100 µm (B) Magnified view of boxed areas of panel A. Lesion margin is demarcated by dotted line. Scale bar, 50 µm (C) “Linearity Index” was quantified for randomly selected cells surrounding a lesion as the ratio of actual junctional length (green) to the linear junction length (straight line between vertices; red). Two sample traced cells and the corresponding linearity index are shown. (D) Linearity index of cells adjacent to lesion is significantly closer to “1” in 8CPT-cAMP-injected eyes. Graph represent average cell linearity index ± SEM, from n = 7 lesions per condition. * p = 1.5763×10*^−^* ^7^.

One mechanism that has been proposed to explain the pro-junctional effects of active Rap1 is enhanced recruitment of junctional proteins and cortical F-actin to sites of cell-cell contact [8], [41]. We examined recruitment of F-actin and β-catenin to RPE cells bordering laser-induced lesions in eyes injected with PBS vs. 8CPT-cAMP. Confocal imaging of these lesions showed an enrichment of junctional F-actin staining in 8CPT-cAMP-injected eyes, particularly in the RPE cells immediately adjacent to the lesion (Fig. 6A–B). Enhanced β-catenin staining and a more linear localization around the cell periphery were also observed in 8CPT-cAMP injected eyes. Changes in cell shape and junctional linearity has been correlated with increased junctional integrity and function [33], [42]. A “junctional linearity index” was calculated as described in Materials and Methods, and representative example cell values are shown in Fig. 6C for visual reference.

RPE cells surrounding the lesions in 8CPT-cAMP injected eyes had significantly greater linearity (i.e. values approaching “1”) (Fig. 6D). These observations suggest that increased recruitment of junctional proteins, F-actin, and enhanced junctional linearity may be a mechanism for the increased barrier properties of the RPE when Rap1 is activated with 8CPT-cAMP.

## Discussion

Our previous study implicated the small GTPase, Rap1, in promoting barrier integrity of an RPE cell monolayer *in vitro* [26]; however, it did not dissect the relative role of each isoform. Furthermore, whether active Rap1 contributed to enhanced barrier function of the RPE *in vivo*, and importantly, whether activation of Rap1A or 1B was relevant to mechanisms relating to choroidal neovascularization, remained to be determined. Using a coculture model relevant to several pathways involved in human AMD [18], [39], [43], [44] and an *in vivo* mouse model of laser-induced CNV, we show that loss of Rap1B *in vivo* results in increased CNV. One possible mechanism for this would be impaired ability of RPE cell-cell junctions to dynamically respond to challenge (i.e. laser-injury) in the absence of Rap1B. Significantly, we can inhibit CNV following laser injury by intravitreal injection of 8CPT-2′-O-Me-cAMP, a compound that activates both Rap1 isoforms. We attribute this effect to the Rap1-induced enhancement of RPE barrier function, via reorganization of cortical F-actin, and possibly the increased recruitment of junctional proteins in cells at the lesion margins.

In endothelial cells (HUVECs), loss of Rap1A significantly reduced impedance and monolayer integrity, and loss of Rap1B had no effect on steady state cell junctional properties [13]. In RPE cells, we show that at steady state, knockdown of Rap1A, but not Rap1B, increased monolayer gap area and decreased TER *in vitro* (Fig. 1), in keeping with these previous observations. In the current study, we also looked at the effects of Rap1 isoform knockdown during dynamic processes such as disassembly/reassembly. Surprisingly, our results revealed that Rap1B shRNA RPE cell monolayers had delayed recovery following washout (Fig. 2A), and were less resistant to EGTA-induced disassembly (Fig. 2B–C), compared to control shRNA or Rap1A shRNA monolayers. We have observed similar Rap1 isoform-specific effects on both steady state and dynamic junctional regulation using primary lung endothelial cells isolated from Rap1a and Rap1b mutant mice (M. Sobczak and M. Chrzanowska-Wodnicka, unpublished data). The distinction between steady state and dynamic junctional regulation is important, as cellular barriers must have intrinsic baseline barrier properties to maintain homeostasis, but must also be primed to respond to various stressors.

We therefore turned to a model in which we could introduce a challenge to the integrity of the RPE barrier. In the laser-CNV model, focal laser-induced thermal and mechanical damage to RPE and Bruch's membrane allows the underlying activated CECs to proliferate, migrate, and invade the retina to form CNV. In the model, CECs can migrate through the lasered region that is void of RPE. In addition, laser induces reactive oxygen species [34], angiogenic [44], [45] and inflammatory factors [46] that compromise barrier integrity of the surviving neighboring RPE, which then cannot resist CEC transmigration into the sensory retina. These events can further increase CNV lesion size. The murine laser-induced CNV model is relevant to human neovascular AMD based on sharing of key molecular mechanisms, and the observation that laser injury can also cause CNV in humans [47]. In AMD, CNV of the normally avascular photoreceptor region of the macula is a major cause of vision loss.

**Figure 7:**
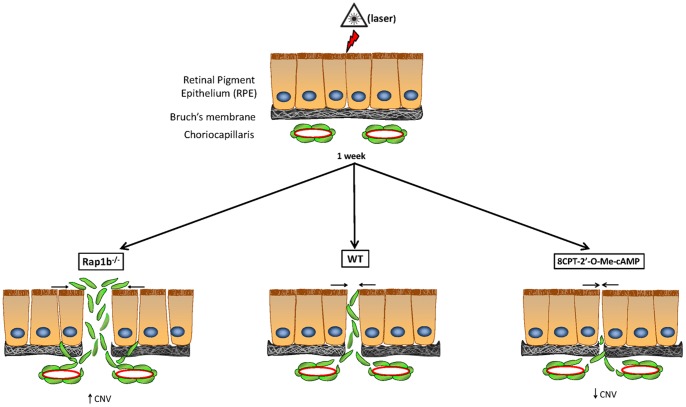
Schematic of cellular events in the laser CNV model. Laser treatment creates a breach in RPE and Bruch's membrane; RPE barrier integrity is compromised in cells adjacent to the lasered region. Inflammatory and wound healing events lead to activation of CECs from the choriocapillaris; CECs begin to migrate and transmigrate the lasered lesion as well as the adjacent RPE with compromised barrier integrity. In Type 2 CNV (shown), CECs proliferate and invade the subretinal space to form CNV. Type 1 (occult) CNV occurs when CECs remain sub-RPE (not shown in this model). 8CPT-cAMP injection post-laser inhibits CNV by promoting barrier integrity in the neighboring RPE, thereby reducing the lesion width through which CECs migrate. CEC junctional integrity may also be strengthened, contributing to the decreased CNV. Compared to WT, *Rap1b^−/−^* RPE cell junctions are more easily disrupted, allowing greater CEC transmigration and increased CNV. 8CPT-cAMP treatment activates both Rap1 isoforms, which is associated with increased junctional resealing and limits RPE monolayer disruption through which CEC migration occurs.

When we induced injury to the RPE by laser photocoagulation and subsequently monitored the neovascular response, we found differences between WT and Rap1a or Rap1b knockout. Following laser, mice lacking Rap1B had significantly greater CNV volume compared with WT and *Rap1a^−/−^* mice (Fig. 3A-B). This result coincides with our *in vitro* studies showing that Rap1B shRNA RPE monolayers were more sensitive to EGTA-induced disassembly, and were slower to re-assemble cell junctions (Fig. 2). Thus, we hypothesized that in *Rap1b^−/−^* eyes the RPE, particularly those adjacent to the lasered regions, are less able to recover from the laser injury and activated CECs can more easily breach the RPE monolayer barrier (Fig. 7 schematic). In the laser-CNV model, day 7 marks the early stages of CNV-complex regression, initiated by proliferation and migration of the RPE over the fibrovascular scar [48]. Containment of migrated CECs in the neovascular tissue [49] would also be aided by re-sealing of RPE cell-cell junctions. CNV volume is a product of both RPE barrier disruption and CEC migration/proliferation into the neurosensory retina. In addition, CECs also have cell-cell junctions, breakdown of which would likewise encourage migration and invasion into the sub-RPE or sensory retinal spaces. Rap1A alone [50], or together with Rap1B [29], [51]–[53], has been shown to be required for efficient cell migration, including angiogenesis, but the results have varied depending on cell type. RPE- and CEC-specific knockout or conditional activation of each Rap 1 isoform in RPE would be required to test in which cell type each isoform plays its more critical role. The involvement of each isoform may depend on cellular context, stimulus, or model system used.

Based on our Rap1 knockout mouse experiments, we hypothesized that conversely, targeted activation of Rap1 in the RPE would provide a novel approach to inhibit CEC transmigration of the RPE and ultimately, reduce CNV. To test the idea that strengthening the RPE barrier could decrease CEC invasion, we utilized 8CPT-cAMP, a chemically modified cAMP analog that can increase the activity of both Rap1 isoforms via activation of the Rap GEF Epac, but does not activate other cAMP-responsive pathways [32]. *In vitro*, treatment of cultured RPE cells with 8CPT-cAMP significantly reduced transmigration of CECs across the cell monolayer (Fig. 5A). We also found that intravitreal injection of 8CPT-cAMP immediately following the laser-injury significantly reduced CNV volumes (Fig. 5B–C). Intraocular injections are the delivery method of choice for traditional AMD therapies targeting VEGF, and are not an unreasonable method of delivery. We have also carefully measured various indicators of toxicity to rule out unwanted side effects (Fig. S3). Other groups have shown that Rap1 activation *in vivo* by methods including 8CPT-cAMP treatment can reduce microvascular permeability [35], [54], protect against renal failure in ischemia-reperfusion injury [55], and inhibit lung vascular leak in ventilator-induced lung injury [12]; our findings suggest Rap1 activation may also be a valid pharmacological strategy to strengthen RPE barrier properties and prevent choroidal neovascularization that occurs in AMD. That being said, activating Rap1 *in vivo* could also strengthen CEC junctions, which could enhance the protective effect of 8CPT-cAMP treatment in inhibiting CNV. CNV can occur either sub-retinal (Type 2), where the RPE layer is breached, or sub-RPE (Type 1, “occult”), where invading CECs remain below the RPE. When visual acuity falls in neovascular AMD, about 50% of the time it is from the transition from Type 1 occult CNV to Type 2 sensory retinal CNV [56]. A junction-promoting effect on CECs would also inhibit either type of CNV.

Once Rap1 is activated, it can then signal to other downstream barrier-promoting signaling pathways in the cell. For example, under certain conditions active Rap1 has been shown to interact with a subset of Rac1 GEFs, such as Tiam1 and Vav2 [57], recruiting these GEFs to the leading edge lamellipodium where they promote localized Rac1 activation and enhanced cell spreading. With respect to barrier function, the Rac1 GEFs Vav2 and Tiam1 have been implicated in increased TER and formation of cortical F-actin following prostaglandin or atrial natriuretic peptide treatment [58], [59]. In contrast, activation of Rho GTPase is associated with dissolution of cortical F-actin structures and production of cytoplasmic stress fibers, with the result being increased myosin-based contractility and increased permeability (reviewed in [60], [61]). Rac1 activation may counteract RhoA activity via redox signaling [62]. Thus, the potential cross-talk between Rap1 and other GTPases such as Rac1 and RhoA is an interesting avenue for future research.

The mechanism for barrier function regulation most often attributed to Rap1 activation is enhancement of junctional protein localization, as well as changes in F-actin distribution. We observed that 8CPT-cAMP treatment promoted an increase in cortical F-actin (Fig. 4A). An increase in cortical actin would provide a means of linking the actin cytoskeleton to cell-cell junctions, and has been associated with the enhanced barrier function of “mature” junctions [10], [35]–[37]. Furthermore, we also observed recruitment of junctional proteins such as ZO-1 and β-catenin to a more continuous, linear pattern both *in vitro* and *in vivo* (Fig. 4). This was particularly apparent in the RPE cells adjacent to the laser lesion (Fig. 6), suggesting an active process of remodeling in these regions. Rap1 is known to bind to multiple junctional proteins, and positive and negative regulators of Rap1 are also recruited to cell junctions (reviewed in [63]). Rap1 undergoes rapid activation during calcium switch experiments when cell junctions are actively assembling or disassembling [9], [64], and biosensor experiments have confirmed that Rap1 is activated at sites of cell-cell contact [65]. These observations hint at a fine-tuning role for Rap1 during situations that require dynamic junction assembly/disassembly. Resolution of RPE barrier function in the laser-CNV model would be an example of this.

The junctional linearization we observe in RPE treated with 8CPT-cAMP both *in vitro* and *in vivo* likely involves non-muscle myosin contractility, through regulation of myosin light chain (MLC) phosphorylation. Many studies have correlated increased phosphorylation of MLC with junctional disruption, increased permeability, and actin cytoskeleton reorganization (recently reviewed in Cunningham *et al*. [66]. In RPE, thrombin-induced phosphorylation of MLC increased actin stress fibers in a Rho kinase and myosin light chain kinase (MLCK)-dependent fashion [67]. Interestingly, Shen *et al*. [68] noted that in epithelia with increased phospho-MLC, cell junctions became “irregularly undulating” which we would characterize as equivalent to having decreased linearity. In corneal endothelial cells, elevating cAMP (which would activate Rap1 in a manner similar to 8CPT-cAMP), helps preserve barrier function after thrombin treatment [69] and also results in MLC dephosphorylation [70]. On the other hand, Nakajima *et al*. found that junction linearization requires localized phospho-MLC; in the absence of this, β-catenin localization becomes discontinuous, and the actin circumferential belt is attenuated [71]. Future studies will determine the phosphorylation status of MLC in RPE treated with 8CPT-cAMP to further clarify the mechanism of junctional linearity.

We interpret our measurement of increased junctional linearity after 8CPT-cAMP treatment (Fig. 6D) as being indicative of uniform circumferential junctional tension between adjacent cells. This is analogous to surface tension-directed packing of soap bubbles and the physical tendency for surface areas to be minimized as in the hexagonal organization of Drosophila retinal cells [72]. Linear geometry, as opposed to a slack or wavy appearance of epithelial cell boundaries has been correlated with increased junctional integrity and barrier properties [33], [42]. In Drosophila, mutation of the Rap1 GEF dPDZ-GEF affected cell shape in wing disc epithelia [73], and more recently, it was shown that Rap1 activity level within a particular subset of cells correlated with this process [74]. Rap1 activity was down-regulated specifically in vein cells, resulting in adhesive asymmetries and non-hexagonal morphology, reminiscent of the non-linear appearance of the cells bordering the lesions of non-8CPT-cAMP treated RPE in our experiments. This also suggests that the increased Rap1 activity in RPE cells treated with 8CPT-cAMP post-laser is playing an important role in directing cell shape changes that promote cell-cell adhesion and junctional conformation.

Rap1 GTPase is becoming appreciated for its role in regulating cell-cell adhesion in both endothelial and epithelial cell systems. *In vitro*, using well-accepted models of epithelial barrier integrity, we show that Rap1A is important in maintaining steady-state RPE barrier integrity, while Rap1B is involved in resisting disassembly and promoting reassembly following junction disruption. Using knockout mouse models, we also provide evidence that Rap1B plays a role in containing and/or preventing choroidal endothelial cell invasion across the RPE and into the sensory retina, a step that is relevant in a number of eye diseases, including choroidal neovascularization associated with degenerative myopia, central serous retinopathy and age-related macular degeneration. In fact, recent computer simulations suggest that impairment of RPE-RPE adhesion is a major contributor in the transition to vision threatening sub-retinal CNV [75], perhaps facilitating the development of sensory retinal CNV by permitting otherwise restricted entry of growth factors and cells into the sensory retina from the choroid. This, together with our data showing that Rap1 activation using compounds such as 8CPT-cAMP reduces CEC migration and CNV suggests that targeting Rap1 may be a promising pharmacological treatment method for neovascular AMD and has potential for application to other human diseases where loss of barrier integrity occurs.

## Supporting Information

Figure S1 **Rap1 isoform shRNA and antibody specificity.** (A) To confirm knockdown specificity, lysates from cultured RPE expressing negative control, Rap1A, or Rap1B shRNA adenoviral constructs were probed with isoform-specific antibodies and β-actin as a loading control. (B) Rap1 isoform antibody specificity. Total cell lysates of lung tissue obtained from WT, Rap1a−/−, or Rap1b−/− mice were run on SDS-PAGE and Western blotted using antibodies against the Rap1A isoform, Rap1B isoform, and total Rap1 (1A+1B) as indicated. For each set, blots were reprobed with anti-β-actin antibodies to confirm equal protein loading.
(TIF)

Figure S2 **Calcium switch: Rap1B shRNA monolayers are more disrupted and slower to recover following washout.** Cells were plated in parallel and treated with EGTA for 30 min, followed by washout for 1 or 3 hr as in Fig. 2. Lower magnification images show overall monolayer disruption and larger cell-free gap area.
(TIF)

Figure S3 **Intravitreal injection of 8CPT-2**′**O-Me-cAMP is not toxic.** (A) Representative fluorescein angiogram showing location of sd-OCT retinal thickness measurements (marked by X) relative to location of retinal vessels and lasered spots (lighter circular regions). (B) Representative sd-OCT cross-section showing retinal layers. (RNFL/GC, retinal nerve fiber layer/ganglion cell layer; IP, inner plexiform layer; IN, inner nuclear layer; OP, outer plexiform layer; ON, outer nuclear layer; ELM, external limiting membrane; IS/OS, inner/outer segment of photoreceptors; RPE, retinal pigment epithelium) Red arrow denotes region of retinal thickness measurements. (C) Quantification of retinal thickness using the average measurements from 2–4 regions per sd-OCT image of n = 18 eyes per condition (PBS vs. 8CPT-2′O-Me-cAMP intravitreal injections). Intravitreal injections of 8CPT-2′-O-Me-cAMP does not increase retinal thickness. (D) Western blot of RPE-choroid cell lysates 24 hrs following intravitreal injection of PBS, 8CPT-2′-O-Me-cAMP, or staurospaurine-treated H1B-1B cells as a positive control, using antibodies for caspase-3 and cleaved-caspase 3. β-actin levels serve as a loading control. (E) TUNEL staining (green) of cryo-sectioned eyes 24 hrs following PBS or 8CPT-2′-O-Me-cAMP injection. DNase-treatment of cryosections served as positive control.
(TIF)
