# Supplementary figures and images for: Rap1 GTPase Activation and Barrier Enhancement in RPE Inhibits Choroidal Neovascularization In Vivo
Source: PLoS One. 2013 Sep 10;8(9):e73070. doi: 10.1371/journal.pone.0073070 (PMC3769400; doi:10.1371/journal.pone.0073070)

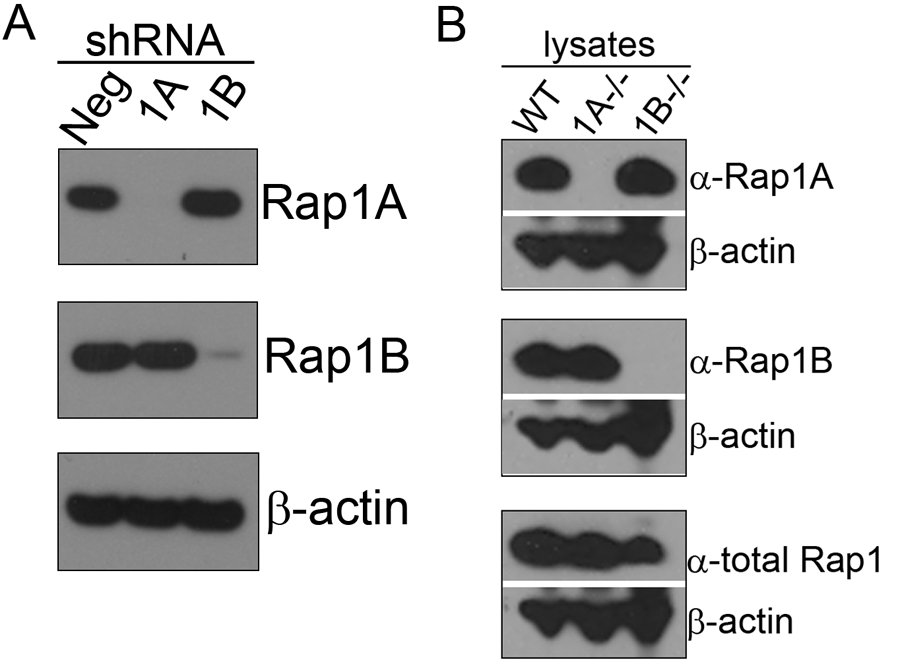

Supplement: Figure S1 — Rap1 isoform shRNA and antibody specificity. (A) To confirm knockdown specificity, lysates from cultured RPE expressing negative control, Rap1A, or Rap1B shRNA adenoviral constructs were probed with isoform-specific antibodies and β-actin as a loading control. (B) Rap1 isoform antibody specificity. Total cell lysates of lung tissue obtained from WT, Rap1a−/−, or Rap1b−/− mice were run on SDS-PAGE and Western blotted using antibodies against the Rap1A isoform, Rap1B isoform, and total Rap1 (1A+1B) as indicated. For each set, blots were reprobed with anti-β-actin antibodies to confirm equal protein loading. (TIF) [file pone.0073070.s001.tif]

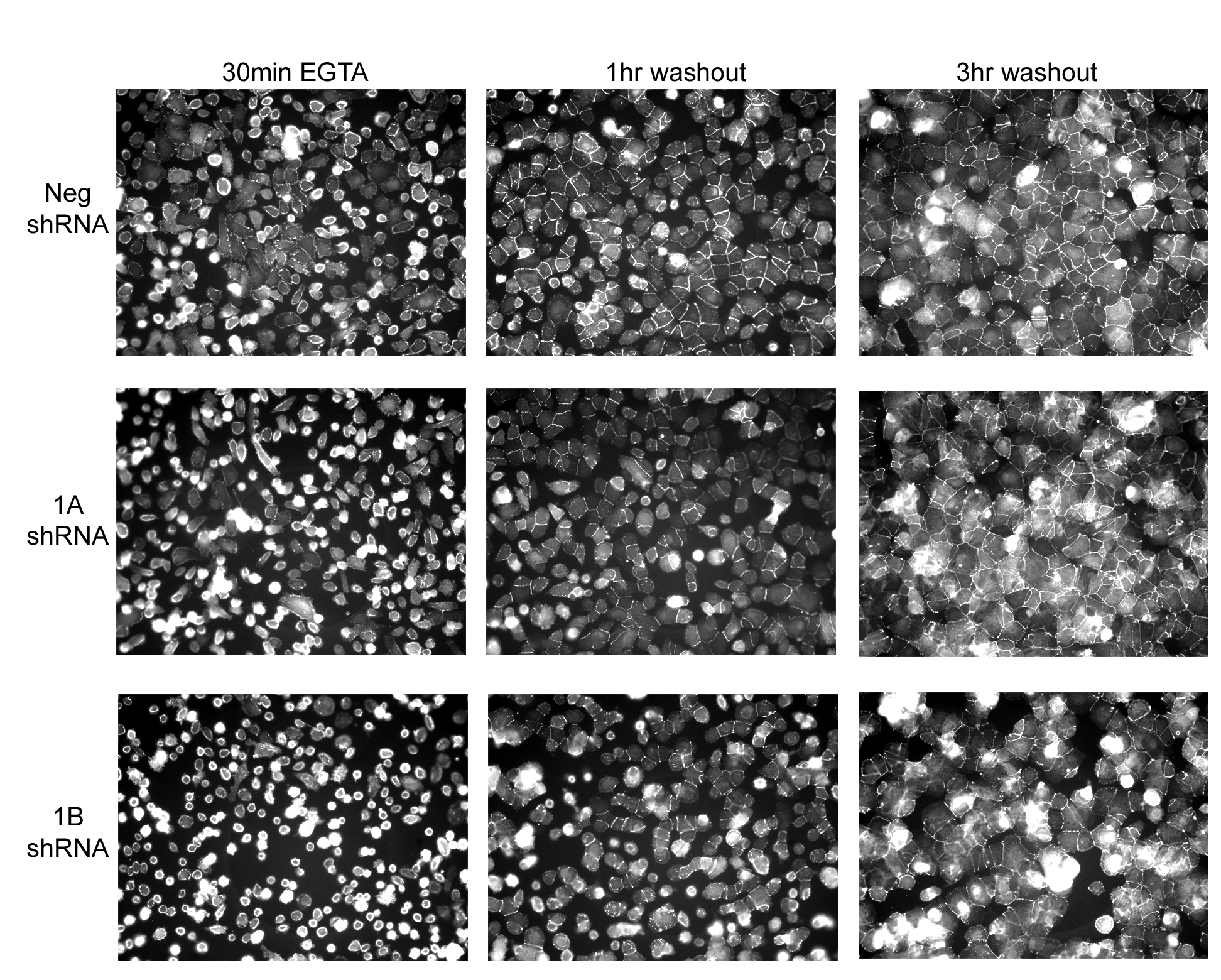

Supplement: Figure S2 — Calcium switch: Rap1B shRNA monolayers are more disrupted and slower to recover following washout. Cells were plated in parallel and treated with EGTA for 30 min, followed by washout for 1 or 3 hr as in Fig. 2. Lower magnification images show overall monolayer disruption and larger cell-free gap area. (TIF) [file pone.0073070.s002.tif]

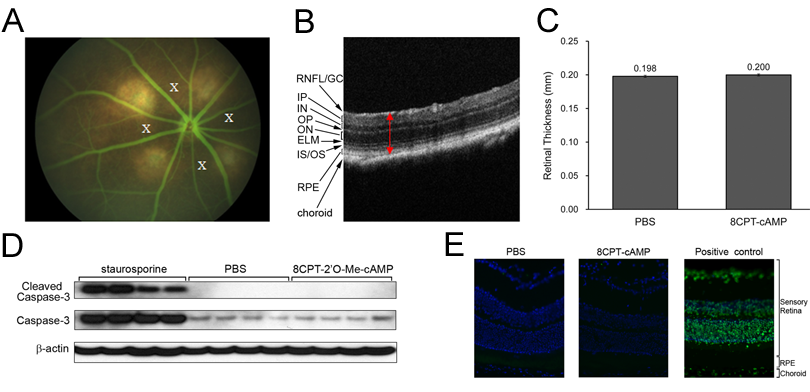

Supplement: Figure S3 — Intravitreal injection of 8CPT-2′O-Me-cAMP is not toxic. (A) Representative fluorescein angiogram showing location of sd-OCT retinal thickness measurements (marked by X) relative to location of retinal vessels and lasered spots (lighter circular regions). (B) Representative sd-OCT cross-section showing retinal layers. (RNFL/GC, retinal nerve fiber layer/ganglion cell layer; IP, inner plexiform layer; IN, inner nuclear layer; OP, outer plexiform layer; ON, outer nuclear layer; ELM, external limiting membrane; IS/OS, inner/outer segment of photoreceptors; RPE, retinal pigment epithelium) Red arrow denotes region of retinal thickness measurements. (C) Quantification of retinal thickness using the average measurements from 2–4 regions per sd-OCT image of n = 18 eyes per condition (PBS vs. 8CPT-2′O-Me-cAMP intravitreal injections). Intravitreal injections of 8CPT-2′-O-Me-cAMP does not increase retinal thickness. (D) Western blot of RPE-choroid cell lysates 24 hrs following intravitreal injection of PBS, 8CPT-2′-O-Me-cAMP, or staurospaurine-treated H1B-1B cells as a positive control, using antibodies for caspase-3 and cleaved-caspase 3. β-actin levels serve as a loading control. (E) TUNEL staining (green) of cryo-sectioned eyes 24 hrs following PBS or 8CPT-2′-O-Me-cAMP injection. DNase-treatment of cryosections served as positive control. (TIF) [file pone.0073070.s003.tif]
